# Identification of a Ferroptosis-Related Prognostic Gene PTGS2 Based on Risk Modeling and Immune Microenvironment of Early-Stage Cervical Cancer

**DOI:** 10.1155/2022/3997562

**Published:** 2022-04-08

**Authors:** Chang Zou, Fangfang Xu, Jiacheng Shen, Shaohua Xu

**Affiliations:** Department of Gynecology, Shanghai First Maternity and Infant Hospital, School of Medicine, Tongji University, Shanghai 200092, China

## Abstract

**Background:**

Cervical cancer (CC) has long been a concern, as a gynecological cancer type of high-risk. At present, there are few studies on the early detection of CC at the genetic level. The breakthrough is to recognize CC patients tending to have a worse prognosis by checking the expression pattern of ferroptosis-related genes, which enjoy a great potential of being applied to cancer treatment.

**Methods:**

Data used in this study was obtained from a series of public online databases, integrated with ferroptosis-related gene collection stored from the FerrDb database and GeneCards database. The least absolute shrinkage and selection operator- (LASSO-) penalized analysis was taken for modeling, and before, univariate Cox regression analysis got done to shrink the candidates' range. Several analyses were made for the evaluation of the efficacy of the new model, based on CC patients' overall survival (OS). Tumor microenvironment- (TME-) related analyses were conducted by various algorithms on different populations, comprising CIBERSORT, ssGSEA, XCELL, etc. Nonnegative matrix factorization (NMF) clustering got applied to find that ferroptosis-marker genes affect prognosis more than “driver” and “suppressor”. Hub-gene PTGS2 was screened out by protein-protein interaction analysis and real-time qPCR after ferroptosis induction, and ELISA was conducted for further verification on the correlation between ferroptosis and M1 polarization.

**Results:**

The twenty-five ferroptosis-related genes model can estimate the prognosis of patients independently of other clinical factors, and the low-risk score group shows higher expression of immune-enhancing cells, noteworthily for M1 macrophages. It is experimentally validated that the M1 marker TNF-*α* significantly increased after coculturing M1 macrophages and SiHa cells processed with ferroptosis inductor before. The key gene to the model, PTGS2, presented to be a risk factor in cervical cancer, and its low-expression group has stronger immune activity and higher tumor mutation burden, with the significantly highly mutated gene TENM2 in it showing high drug sensitivity and neoantigen for patients with its mutant-type. Meanwhile, it influences macrophage polarization.

**Conclusion:**

Prognosis of early-stage cervical cancer patients can be exactly predicted on ferroptosis-related genes. Among model genes, PTGS2 may have a major impact by affecting macrophage polarization and mutation effects.

## 1. Introduction

Cervical cancer continues to be a threat to the health of middle-aged women, particularly in countries with backward healthcare facilities [1]. In 2018, there were approximately 569000 CC cases diagnosed and it caused probably 311,000 dead cases worldwide. It remains the status ranking fourth in the list of most fatal malignant tumors for global women [2]. Besides the high morbidity, there is a poor prognosis for CC patients. The rate of failing to achieve complete response for patients with locally advanced disease (FIGO stage IIB-IVA) receiving concurrent standard treatment (CCRT, with cisplatin alone or in combination) reached about 30%-40% [3].

Ferroptosis was discovered as a new type of programmed cell-death, which was differently defined from necrosis and apoptosis and prompted by iron-dependent lipid peroxide accumulation. The cytological feature for recognition of ferroptosis in cells is inclusive of cell volume reduction and mitochondrial membrane density increasing. It can be triggered by substances in low-molecular or drugs which function as system X (c) inhibitors or glutathione peroxidase 4 (GPX4) inhibitors, such as erastin, artemisinin, and its derivatives, sorafenib. Ferroptosis had been reported to exert an unneglectable influence on some types of tumor growth, such as lymphocytes, RCC, and HCC [4–6]. Cancer cells show an increased demand for iron to meet the need for growth and proliferation, this iron dependency always creates a satisfying response to treatments based on iron-catalyzed necrosis, that is what makes ferroptosis kill tumor cells efficiently. The evasion of tumor cells to cell death execution still remained a challenge for therapy, and FDA-approved drugs recognized as ferroptosis inducers can probably bring a new breakthrough [7].

Cervical cancer is not only with high-risk and rapid progress but also insidious and always without symptoms. About two-thirds of CC patients had advanced to locally advanced cervical cancer when being diagnosed. Even with correct multidisciplinary management, these patients probably ended with a low survival rate [8]. If there can be an effective predictive index designed on the gene expression pattern of early-stage CC patients, to screen for the susceptible population to CC in advance from the genetic level, the curative ratio of early-stage cervical cancer may be significantly improved. As mentioned above, ferroptosis may enhance the effect of therapies and become an important way to kill cancer cells. However, currently, there are few studies on the correlation between ferroptosis and the occurrence and development of CC cells, as well as the prognosis of cervical cancer patients.

**Figure 1:**
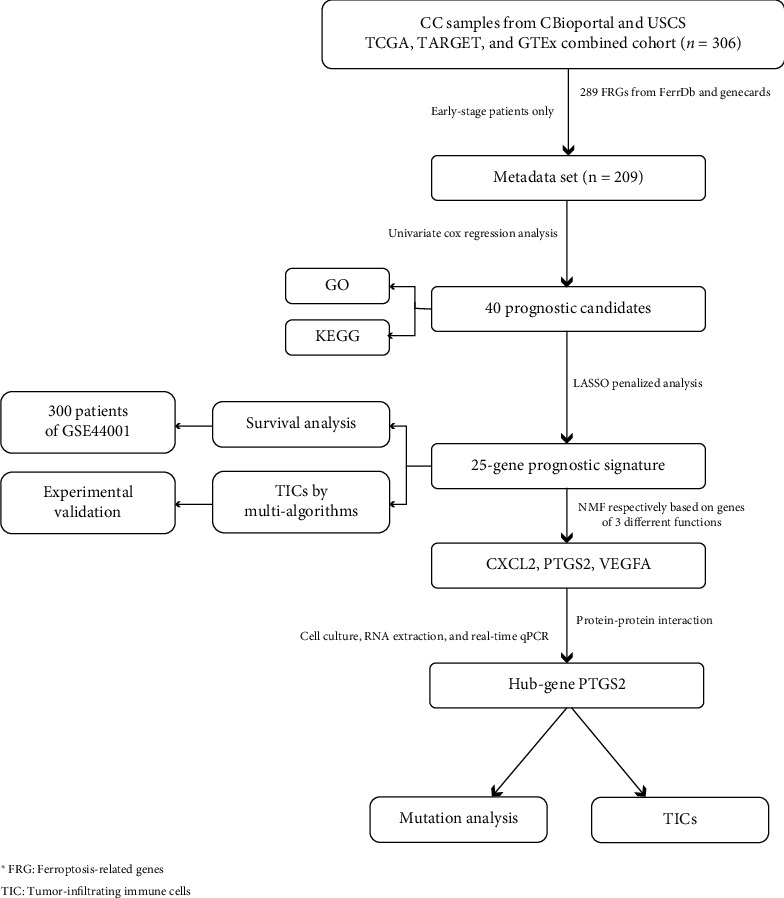
The flowchart.

In this study, we observed a new prognostic signature on 25 ferroptosis-related genes (FRGs). The model was established on integration of expression data from TCGA-CESC cohort and ferroptosis-related genes from FerrDb [9] and GeneCards database, through the univariate Cox regression combined with LASSO-penalized analyses and validated in the microarray dataset GSE44001 from the GEO dataset. A set of independent prognostic analyses got done to confirm the reliability of the biomarkers. We scored the risk of tumor samples with the new-built model and compartmentalized the early-stage CC patients to groups inferring to the median of the risk score as a cut-off, to further explore how the FRG expression pattern immanently affected the prognosis of CC. The results revealed the relationship between FRG expression and tumor immunity and tumor microenvironment infiltration, providing new ideas for the application of ferroptosis in the field of CC. When compared with the model from other research, this one enjoyed superiority in accuracy. Nonnegative matrix factorization clustering was conducted on expression submatrix partitioned by the role of genes in ferroptosis; thereafter, we figured out that ferroptosis-marker genes contributed most to the prognosis of CC patients. As the most significantly increased marker-gene among model features after ferroptosis induction, analyses were launched around key-gene PTGS2. The low-PTGS2 group enjoyed a higher percentage of M1 macrophages, with a higher tumor mutation burden, and the mutation of the most significantly different mutated gene, TEMN2, seemed to contribute to the efficacy of immunotherapy, for having a potential correlation with high neoantigens and PD-L1 high-expression. Through real-time qPCR and ELISA, we suggested the secretion level of M1 marker TNF-*α* positively correlates with ferroptosis. Complete information about the process was exhibited in Figure 1.

## 2. Materials and Methods

### 2.1. Data Resources

RNA sequencing data provided by a combined cohort of TCGA, TARGET, and GTEx samples from UCSC Xena website (https://xenabrowser.net/) and 306 CC patients' genetic information got extracted (Table 1). Clinical characteristics including grade, overall survival data, HPV infection, clinical stage, and the age at initial diagnosis were downloaded from the GDC TCGA CESC cohort also on UCSC and cBioportal (http://www.cbioportal.org/). FRGs were collected from the GeneCards database (https://www.genecards.org/) by searching the keyword “ferroptosis,” and selectively choosing genes with high relevance, and the FerrDb database (http://www.zhounan.org/ferrdb/) by downloading in CSV form. The FRGs stored from the FerrDb database can be compartmentalized into three subtypes: driver, suppressor, and marker, and depending on their roles in the ferroptosis process, the reliability of their correlations with ferroptosis were divided into four confidence levels, from high to low as validated, screened, predicted, and deduced. After the integration with existing materials, complete expression data of 281 ferroptosis-related genes were obtained. The microarray dataset GSE44001 for external validation consisting of 300 patients with early-stage CC (Stage I or II) was acquired from the GEO database [10] (https://www.ncbi.nlm.http://nih.gov/geo/).

In order to focus the study on patients with early cervical cancer, samples meeting the following criteria were removed: (1) survival period < 30 days; (2) with incomplete clinical information; [4] the cause of death had nothing to do with CC; (4) healthy samples; and (5) clinical stage III or IV. Thus, 209 samples were up for further analyses, see Table 1 for details. The estimation on the immune cell infiltration of samples from TCGA was acquired from the websitehttp://timer.cistrome.org/(TIMER 2.0), and the following analyses on the somatic mutation of CC cases were based on information stored directly from the TCGA website (https://portal.gdc.cancer.gov/).

### 2.2. Construction of Prognostic Signature for Early-Stage CC Patients according to FRGs

#### 2.2.1. The Model Construction

“Survival” package by R was applied to conduct a univariate Cox regression analysis, with the range of *p* value < 0.05, which showed genes that were significantly correlated with the prognostic survival of CC patients among the 281 FRGs. Later, a 10-folded LASSO-penalized analysis was made via the “glmnet” tool [11, 12] to screen for genes whose expression level works as the main risk characteristics that contribute most to the prognosis. We derived the regression coefficients of selected candidate genes whose expression data were taken into the calculation and figured out risk scores of CC patients according to the following formula: risk score = expression × coefficient of gene 1 + expression × coefficient of gene 2 + expression × coefficient of gene *n*. The model got verified by having the datasets from GEO going through the same formula. We took the median value of the risk scores as a standard to compartmentalize CESC patients into low- and high-risk clusters.

#### 2.2.2. Validations on the Accuracy of the Prediction

Obviously, unlike advanced ones, there are few effective methods for patients with early-stage tumors to assess the risk of their current disease; thus, we hoped risk score can be considered as a new-found potential prognostic indicator of CC. To see the accuracy of its function of prediction, risk core was taken as a variable juxtaposed with other clinical features, including clinical stage, neoplasm histologic grade, HPV infection, and age, and analyzed with Cox regression model in both multivariate and univariate ways. ROC (time-dependent receiver operating characteristic) and DCA (decision curve analysis) were shown in curves, made to compare risk score with other clinical indicators. We chose the survival data on the 1st, 3rd, and 5th years as time nodes for the analysis. Meanwhile, we also collected models with a similar function of predicting CC patients' prognosis from other researches and scored the samples we used to model; then, ROC curves were put into comparison to show the superiority of our new-built model.

### 2.3. GO and KEGG Enrichment Analysis

Gene Ontology (“ GO ” in short) and Kyoto Encyclopedia of Genes and Genomes (“ KEGG ” in short) analyses got operated to reveal the enriched signaling pathways. Thanks to R packages “clusterProfiler,” “enrichplot,” and “ggplot2.” Pathways meeting the standards below got taken as significantly enriched: (1) *p* value < 0.05 and (2) *q*-value < 0.05.

### 2.4. Survival Analysis

The original materials for survival analysis were overall survival data extracted from the phenotype data on the UCSC Xena website, of the CC cohort on GDC TCGA. All the survival analyses of this study got accomplished by R “survminer” package [13]. Results with a range of *p* value < 0.05 got taken as having significance.

### 2.5. Analyses about Immune Features on Groups Divided according to Risk Scores

Single sample GSEA (ssGSEA) was conducted with the “GSVA” R package [14], while immune-gene-sets got obtained from the database called MSigDB (https://www.gsea-msigdb.org/).The ESTIMATE algorithm with R software [15] was adopted to evaluate the components of the TME and stromal-immune proportion of each CC sample, and the results were converted into 3 numeric indicators as immune score, stromal score, and ESTIMATE score, respectively. The larger the value, the larger the proportion of the corresponding microenvironmental component. The information of the tumor-infiltrated immune cells proportions of TCGA samples was stored from the online database, the proportions of immune cells were figured out via several algorithms inclusive of XCELL, MCP-counter, quanTIseq, and EPIC, and all the calculation process got done by TIMER 2.0 [16] and R-software tool (version 4.0.5). According to results obtained by the algorithms mentioned above, we made an analysis on the differential expression of immune cells between different risk-degree clusters, those with a significant difference were displayed altogether by package “pheatmap” in the R tool. The same analysis was made on common immune checkpoint (ICP) expression. All the analyses above suggested that, in general, the low-risk group tended to enjoy a relatively more active immune microenvironment. Packages “limma,” “ggplot2,” and “ggplot” [17] were adopted when visualizing all the different distributions.

When it came to the calculation result of the differential analysis based on the CIBERSORT scoring system [18], there was a noticeable fact that macrophage M1 presented a significantly higher expression level in the low-risk cluster while M2 expressed similarly between the two groups. We supposed that the expressing pattern of genes involved in model construction exerts a certain influence on macrophage polarization. In order to further explore the possible correlation between model genes' expression pattern and the ferroptosis of CC cells and the macrophage polarization and target a key gene with the most contribution for further discussion, we designed and conducted the following experiments.

### 2.6. Nonnegative Matrix Factorization (NMF) Clustering

The 281 ferroptosis-related genes expression matrix was made into 3 partitions according to gene function called driver-group, marker-group, and suppressor-group, respectively. These function-based cohorts subsequently underwent NMF clustering one by one with the R package NMF, and comprehensive correlation coefficient was calculated to decide *k*-value as the optimal one. Samples in the matrix will be set into clusters, and the quantity of the subgroups will be the best k. The consensus matrix was displayed in the heatmap to see whether the boundary between subgroups was clear and sharp. If so, we would compare the survival between the subgroups and select those with significant between-subgroup differences for further analysis.

### 2.7. Cell Culture, RNA Extracting, and RT-qPCR

The emptions of CC human cell lines SiHa, as well as leukemic cell line of human, THP-1 cells, were from the ATCC agent (Manassas, VA, USA). Afterward, SiHa cells were conserved in DMEM/High Glucose culture medium (Servicebio, China) supplied by 10% fetal bovine serum (FBS, Biological Industries, Israel), humid incubator containing with 37°C temperature control and 5% CO_2_ available, having 1% penicillin/streptomycin (New Cell & Molecular Biotech Co, China) served, and THP-1 cells were kept in RPMI 1640 medium. The THP-1 cells (5 × 104 cells/100 *μ*l) were sprinkled in a 6-well plate (Corning, USA), which was observed to diverge into macrophages, after adding 100 ng/ml PMA (MCE, China) and keep it for 48 hours. After differentiation towards M0 macrophages accomplishing, 100 ng/ml lipopolysaccharide (LPS) (Peprotech, USA) was added, with which macrophages were cultured for 48 hours, then successfully differentiated to M1. A transwell device (Corning, USA) with a 0.4 *μ*m porous membrane got applied for the coculture operations. Before coculture, the SiHa cells were treated with different concentrations of erastin (10 *μ*M and 20 *μ*M, HY-15763, MCE) for 24 h. Then, SiHa cells were planted on the upper chamber of the Transwell device, when M1 were planted with a density as 2 × 10^5^ per well. When the 24-hour-coculture is finished, SiHa cells and the M1 got harvested for the next step, and the culture supernatants were also stored for detecting the secretion level of TNF-*α*.

Besides, we extracted the total RNAs from SiHa cells with TRIzol (Invitrogen, USA); followingly, we evaluated the purity quotient as well as concentration and then launched a reverse transcription towards cDNA, via 5X ALL–IN-One RT Master Mix kit (Applied Biological Materials Inc, Canada). Real-time PCR was also performed using TB Green Premix Ex Taq kit (Takara, Japan), with housekeeping GAPDH considered as the internal control. The primers used in this study were completely listed in Table 2.

A PPI (protein-protein interaction) analysis was conducted via Cytoscape software (V3.8.2), showing the relation network among the products of model genes [19]. The key candidates were screened out with criteria as degree > 5. “Cytohubba” tool in Cytoscape produced a marked effect.

### 2.8. Quantification of TNF-*α* by ELISA

A commercially available ELISA kit (Mlbio, China) was used to determine TNF-*α* secretion levels from the culture supernatants of macrophage M1 after coculturing with SiHa cells under different erastin concentrations. Samples were measured in 3 biological replicates. All experimental steps were performed according to the manufacturer's instructions, and the optical density (OD) was measured at 450 nm. All of the statistical analyses and graphs were performed by GraphPad Prism 8.

### 2.9. Mutation Related Analyses

Somatic mutation data of CESC cases got gained from TCGA in MAF. The mutation scores of samples computed from the following algorithm were taken as Tumor Mutation Burden (TMB): (total mutation/total covered bases) × 106. Gene mutation spectrum and the comutation distribution of CC samples were depicted in oncoplot, forest plot, and heat map via the MAFTOOLS package [20]. Web tools provided by the CAMOIP website (https://www.camoip.net) were applied for immunogenicity, immune checkpoints, and drug sensitivity analyses.

## 3. Results

### 3.1. The Workflow

We constructed a prognostic model containing 25 FRGs. The model was established on the integration of the expression data of 306 CC samples from the TCGA-CESC cohort and the comprehensive FRG list from the FerrDb and GeneCards databases. The univariate Cox regression combined with the LASSO penalty analysis was for modeling, and the outcome formula got validated in array dataset GSE44001. Information about FRGs participated in the study is listed in Table 3. We score the risk of CC samples, early-stage CC patients got partitioned into two cohorts, with the median of the risk scores (value = 5.586) as the demarcation point to further explore how FRG expression patterns affect tumor- immunization and TME infiltration. After performing NMF clustering on the expression submatrix divided by the role of genes in ferroptosis, we found that “marker” genes matter most to the prognosis of CC patients. As the most significantly increased marker gene in the model characteristics after ferroptosis induction, analyses were carried out around the key-gene PTGS2.

### 3.2. The Enriched Functional Pathways of FRGs Contributed to Prognosis

**Figure 2:**
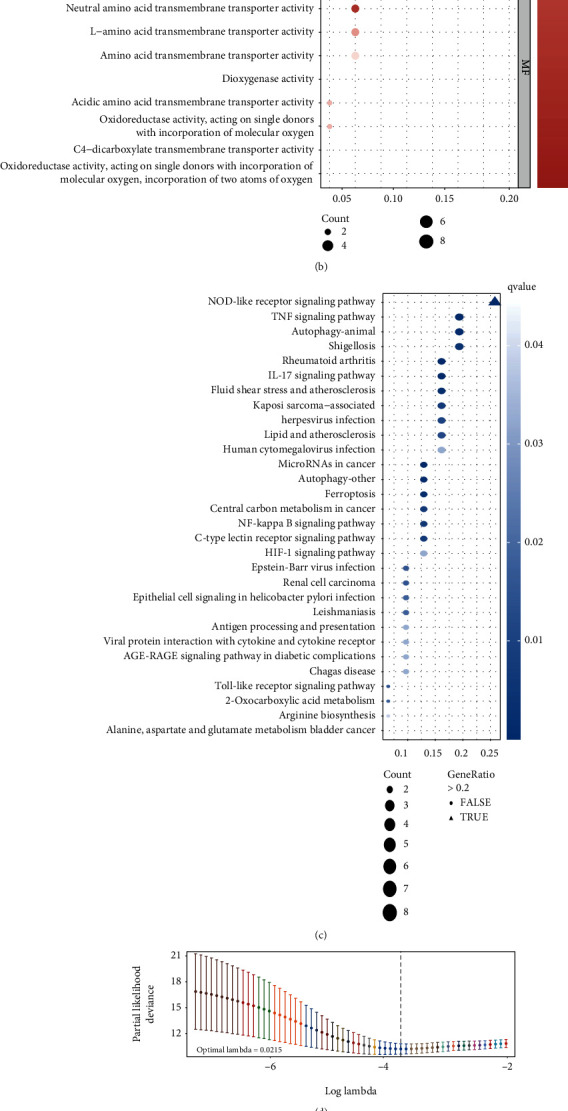
(a) Forest plot depicts 40 prognostic FRGs and the hazard ratios inferred from the outcome of univariate Cox regression analysis, 20 risk factors are marked in yellow, 20 protective factors in blue. (b) and (c) The top 10 pathways among biological process, cellular component, and molecular function in GO analysis (b), and top 30 in KEGG analysis (c) were shown in bubble plot, respectively, the bubble with gene-ratio > 0.2 is triangle shaped. (d) and (e) The least absolute shrinkage with 10-folded cross-validation, conducting candidate shrinkage to build the model. (f) and (g) The univariate and multivariate connections of risk score and other common clinical factors with OS. (h) DCA curves show that the risk score has the longest distance to the basic line. (i) Time-dependent ROC curves show the efficacy on prediction of the new model with AUC = 0.791 > 0.640.

As mentioned above, a univariate Cox regression analysis was made; thus, the 40 FRGs showed their significant correlation with prognosis with a limited range of *p* value < 0.05.20 of them represented a better survival according to this result, while the expression data of another half had the potential of being high-risk indicators. (Figure 2(a)). According to the results for the GO enrichment analysis, genes' functions about the biological process (BP) were closely related to the biochemical processes contributed to ferroptosis, and those about molecular function (MF) were tightly centered around amino acid transportation and oxidoreductase activity. The result above suggested that most of the gene-enriched functions served the occurrence of ferroptosis (Figure 2(b)). Simultaneously, the gene-enriched signaling pathways revealed by KEGG analysis got displayed in descending order of the gene ratios, and besides ferroptosis, it is also inclusive of NOD-like receptor signaling pathway, TNF signaling pathway, autophagy, etc. (Figure 2(c)).

### 3.3. A Prognostic Model Established on LASSO-Penalized Analysis

We sent the 40 prognostic FRGs for shrinkage by LASSO-penalized analysis to concentrate on those risk characteristics that contributed the most to the prognosis of CC patients. The independent variables (candidate signatures) can be screened, and a better fit was explored with a constraint condition which was to the sum of the absolute values of the coefficients, to achieve the purpose of data dimensionality reduction. After the analysis with ten-folded cross-validation, a 25-gene prediction model having its candidate gene-set will function on the sum of the products of their expression values and corresponding regression coefficients, respectively (Figures 2(d) and 2(e)), as shown in Materials and Methods Section. The expression data of genes survived the LASSO analysis were then put into risk calculation: TF (transferrin), TFRC (transferrin receptor), SLC1A5 (solute carrier family 1 member 5), ATG5 (autophagy related 5), WIPI2 (WD repeat domain, phosphoinositide interacting 2), PRKAA2 (protein kinase AMP-activated catalytic subunit alpha 2), TNFAIP3 (TNF alpha-induced protein 3), TAZ (tafazzin), PTGS2 (prostaglandin-endoperoxide synthase 2), JDP2 (Jun dimerization protein 2), VEGFA (vascular endothelial growth factor A), CXCL2 (C-X-C motif chemokine ligand 2), SLC2A8 (solute carrier family 2 member 8), STMN1 (stathmin 1), ISCU (iron-sulfur cluster assembly enzyme), JUN (Jun protooncogene), TMBIM4 (transmembrane BAX inhibitor motif containing 4), MDM2 (MDM2 protooncogene), IDH2 (isocitrate dehydrogenase (NADP(+)) 2), SLC11A2 (solute carrier family 11 member 2), DECR1 (2,4-dienoyl-CoA reductase 1), TNF (tumor necrosis factor), EGLN1 (Egl-9 family hypoxia inducible factor 1), ATG3 (autophagy related 3), and SLC1A4 (solute carrier family 1 member 4).

The result figured out by formula below will be called risk score, which is proportional to the after risk of a CC patient: risk score = (CXCL2 expression × 0.01757) + (TFRC expression × 0.02259) + (PTGS2 expression × 0.03608) + (TNF expression × 0.05541) + (PRKAA2 expression × 0.08078) + (ATG5 expression × 0.13071) + (TNFAIP3 expression × 0.19564) + (JDP2 expression × 0.28366) + (TMBIM4 expression × 0.40741) + (EGLN1 expression × 0.42211) + (JUN expression × 0.45560) + (SLC11A2 expression × 0.60653) + (WIPI2 expression × 1.14623) − (TF expression × 0.06828) − (ISCU expression × 0.63842) − (IDH2 expression × 0.45592) − (ATG3 expression × 0.30001) − (TAZ expression×−0.23449) − (DECR1 expression × 0.20942) − (STMN1 expression × 0.18136) − (SLC1A4 expression × 0.17422) − (SLC2A8 expression × 0.15226) − (SLC1A5 expression × 0.11532) − (MDM2 expression × 0.09991) − (VEGFA expression×−0.08179).

### 3.4. Validation

The outcomes of the univariate (*p* < 0.001, hazard ratio = 3.962, 95% confidence-interval: 2.808–5.589) (Figure 2(f)) and multivariate (*p* < 0.001, hazard ratio = 4.148, 95% confidence-interval: 2.906–5.921) (Figure 2(g)) Cox regression analyses proved that the risk score calculated from FRG-based model works as an independent prognostic indicator for early-stage CC patients. The DCA curve is shown in Figure 2(h), and it turned out that risk score functioned as the only reliable clinical characteristic to be a prognostic signature for our test group. Meanwhile, we also collected a model with similar function of predicting CC patients' prognosis from other research [21], with its predicting formula: (0.195 × TFRC expression values + 0.104 × ACACA expression values + 0.097 × SQLE expression values–0.512 × PHKG2 expression values), and scored the samples we used to model; then, ROC curves were put to comparison to show the superiority of our new-built model (Figure 2(i)).

**Figure 3:**
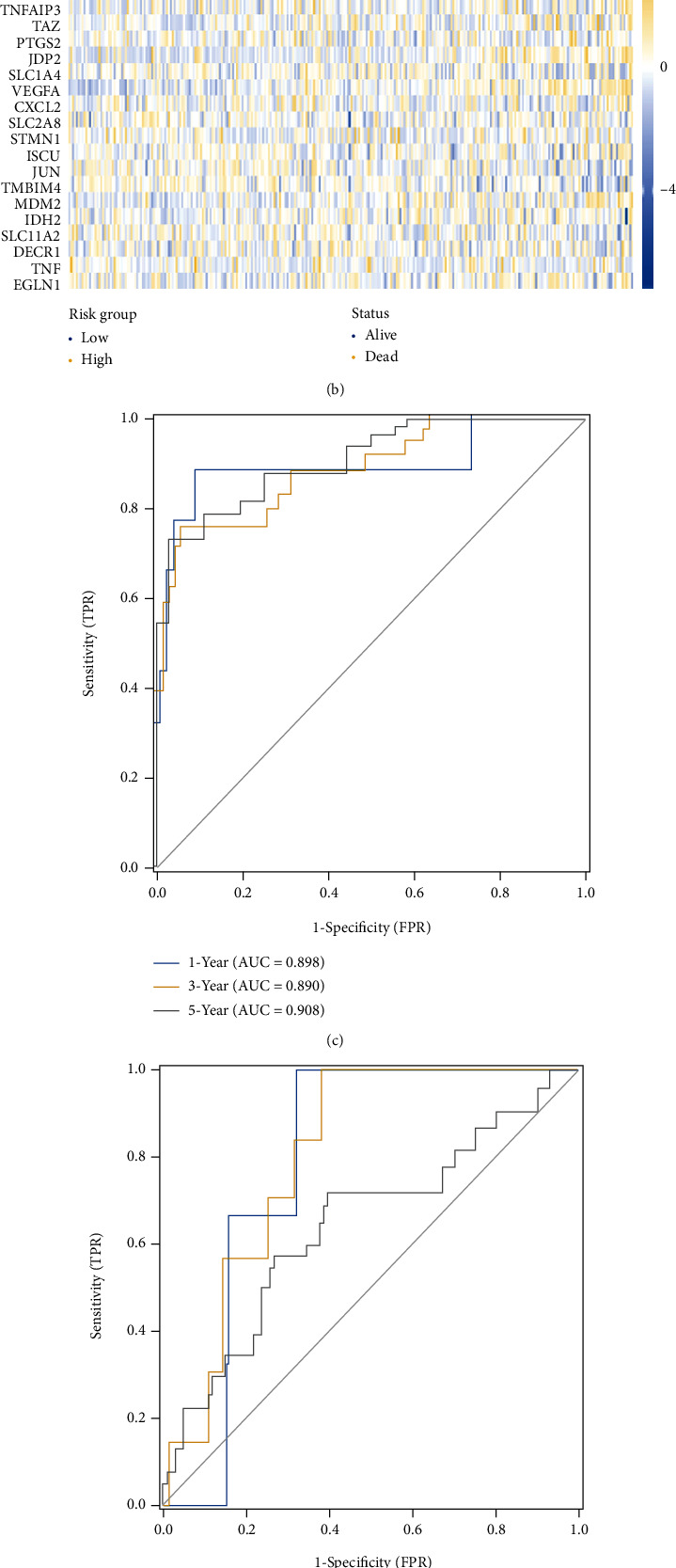
The risk-score distribution, expression heatmaps (a, b), time-dependent ROC curves on 1, 3, 5 year (c, d), as well as Kaplan-Meier curves (e, f) of USCS cohort and external validation GSE44001 dataset are exhibited.

209 CC patients from TCGA were sorted by their risk score and displayed with corresponding survival status in the heatmap, so did 300 samples from the GEO validation dataset, after being scored by FRG-based model and compartmentalized into different risk-degree cohorts with the median value of risk scores (value = 5.235) as a cut-off. The expression distributions of the model genes were also shown in the heatmap; thus, the difference in the distribution between two different risk clusters of every single gene can be clearly seen (Figures 3(a) and (b)). Time-dependent ROC curves were depicted to show the predictive efficacy of the model in both datasets, and the areas under the curve of TCGA data of time nodes are 0.898 (the 1st year), 0.890 (till 3 years), and 0.908 (till 5 years) (Figure 3(c)). For the GEO cohort, the prognostic efficacy got validated by the AUC of 0.789 (the 1st year), 0.804 (till 3 years), and 0.645 (till 5 years) (Figure 3(d)). Besides, the low-risk cluster enjoyed significantly better survival than the high-risk score one according to the outcome of Kaplan–Meier survival analyses to TCGA as well as GEO sets [*p* < 0.001 (Figure 3(e)); *p* = 0.021 (Figure 3(f))]. The outcome attested that when put to be applied in an external dataset, the new-built prognostic model still worked.

### 3.5. Contrast between High- and Low-Risk Score Clusters about Immune-Related Features

#### 3.5.1. Immune-Related Checkpoints

**Figure 4:**
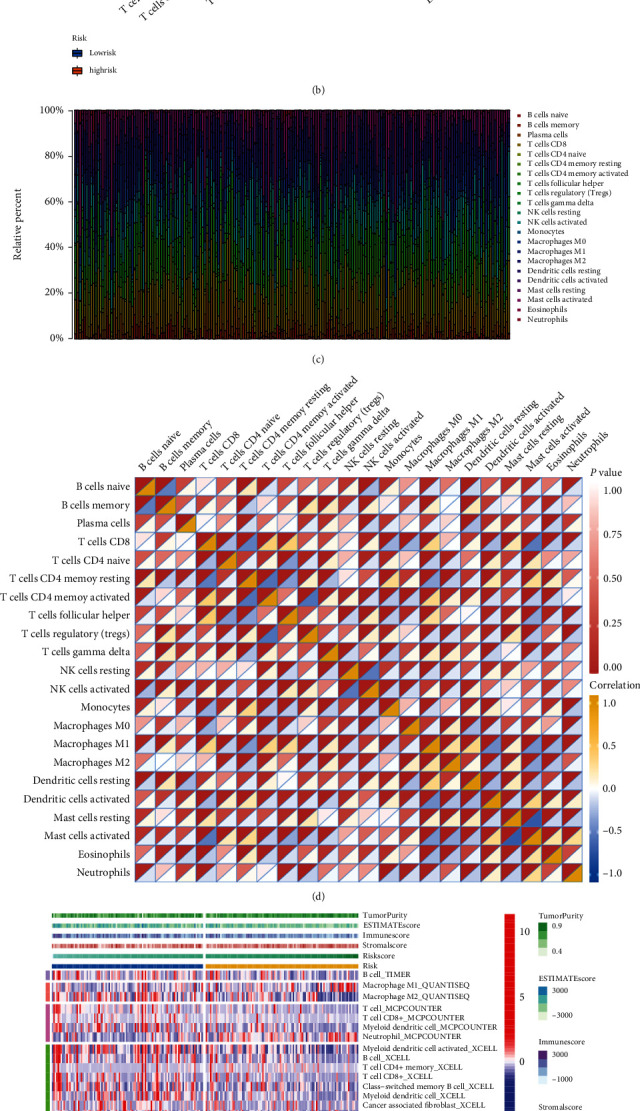
(a) Discrepantly distributed ICPs between clusters with different risk degree, ^∗∗∗^*p* < 0.001, ^∗∗^*p* < 0.01, ^∗^*p* < 0.05. (b) 22 TIC expressions were taken into a comparison between groups of different risk degree via Wilcoxon rank-sum, the outcome was shown in violin graph. (c) The bar plot presents the proportion of TICs of 22 kinds by showing multicolored stripes with different proportions of colored lengths. Sample IDs were set as stripes' names. (d) Heatmap shows the correlation (blue to yellow from low to high) and corresponding significance (presented by the depth of red color at the top left corner) between 22 types of TICs in each box. (e) Multialgorithm immune comprehensive analysis results, inclusive of existing data from platform TIMER, XCELL, MCP-counter, quanTIseq, and EPIC. The top bars show the TME-score distribution corresponding to risk score from low to high. (f) The calculation result of ssGSEA pictured by box plot, ^∗∗^*p* < 0.01, ^∗^*p* < 0.05.

Figure 4(a) shows us the expression distribution of every immune checkpoint (ICP) among the common ones we choose to analyze with significant correlation with the level of risk scores, including BTLA, TNFRSF 14, 18, and 25, CD48, CD276, CD27, and LAG3. Corresponding *p* values were also shown in the picture. In a high-risk cohort, a new-found checkpoint NRP1 (neuropilin-1) was reported as an immune response suppressor to cancer gathered higher expression [22], as well as CD276 [23], which was proven to participate in tumor immune evasion in HNSCC. The ICPs in addition to the above two points expressed more actively in the low-risk cohort. Whether the immunotherapy received by each patient effectively inhibited the checkpoints that contributed to the occurrence and development of tumors may affect the prognosis.

#### 3.5.2. Immune Cell Infiltration Analysis

TIC proportion calculated via different algorithms got simultaneously obtained from the TIMER2.0 website and the outcome of analysis made by R packages CIBERSORT and ssGSEA. A noteworthy fact existing in the outcome of CIBERSORT is that macrophage M1 expressed more actively in the low-risk cohort while macrophage M2 showed no significant discrepancy between the two clusters. Reasonable speculation got made that the macrophage polarization developed differently in low- and high-risk clusters. According to the information above, we made a comparison between two risk-cohorts to see how immune-related components distribution varied. The interesting part of the result was selectively exhibited (Figures 4(c)–4(d)). After the multialgorithm comprehensive analyses, types of immune cells enriched in low-risk group are inclusive of B cell according to TIMER, macrophage M2 in QUANTISEQ, CD4+ memory T cell, CD8+ T cell, and myeloid dendritic cell in MCPCOUNTER, myeloid dendritic cell activated, B cell, T cell CD4+ memory, T cell CD8+, class−switched memory B cell, myeloid dendritic cell, cancer-associated fibroblast, hematopoietic stem cell, B cell memory, T cell NK, and T cell CD4+ Th1 for XCELL, as well as macrophage to EPIC. Obviously, no matter via which method, the low-risk cluster generally presented a higher level of immune activity, such as T and B cells, myeloid dendritic cells, macrophage cells according to TIMER data (Figure 4(e)), as well as immune-related pathways (APC coinhibition, APC costimulation, checkpoint, HLA, T cell coinhibition, as well as costimulation) to the result of ssGSEA analysis (Figure 4(f)). By any method, the result stated that neutrophils were intended to become enriched in the high-risk cohort.

**Figure 5:**
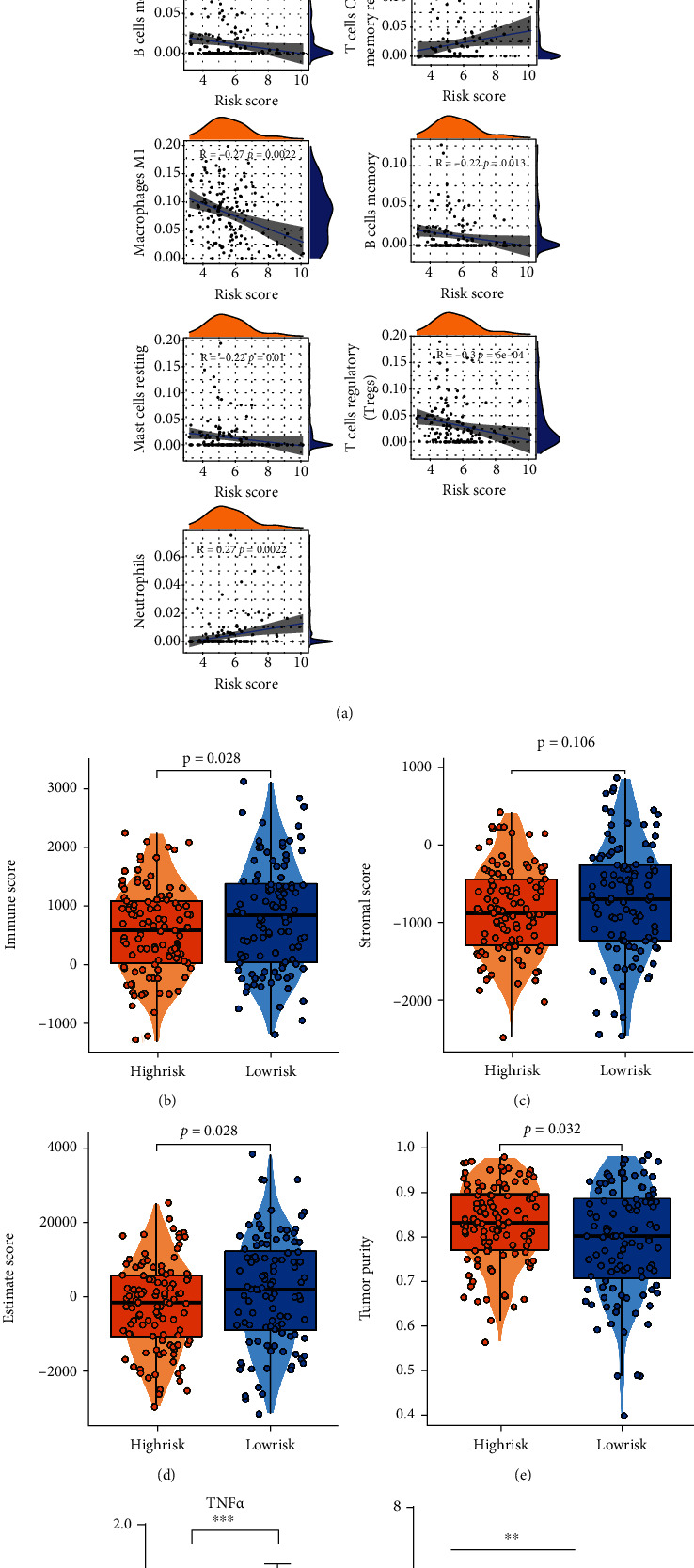
(a) Correlation analyses were made between the risk score and the infiltration of immune cells. The hill-shaped symbols on the top and right imply the density of the corresponding risk score or immune scoring. (b)–(e) The distributions of ESTIMATE-related scores and tumor purity were shown in box plots, low-group with 104 samples while high-group with 105, calculated by Weltch *T*-test. (F) The M1 biomarker TNF*α* increases after ferroptosis-induction (*p* < 0.001). (g) The result of ELISA test, SiHa-NC group with no addition of erastin, SiHa-Fe-Low group added with 10 *μ*M erastin, SiHa-Fe-High group added with 20 *μ*M erastin. TNF*α* secretion level was promoted by the increase of erastin concentration significantly (^∗∗^*p* < 0.01, ^∗^*p* < 0.05).

ESTIMATE algorithm was put into use to assess the TME of CC patients. The distribution of immune, stromal, and ESTIMATE score was shown by the top bar of Figure 4(e), also taken as an immune feature and compared between the two risk groups. The correlation analysis between TICs and risk score was made to enhance the conclusion that the risk degree is connected with thriving M1, as well as CD8+ T cell, the commonly considered contributing ones to immune activity (Figure 5(a)). The result suggested that among all three indicators assessed with the Weltch *T*-test, immune score showed significant difference with *p* value 0.028 (Figure 5(b)), Stromal-Score with *p* value = 0.106 (Figure 5(c)), ESTIMATE-Score with *p* value = 0.028 (Figure 5(d)), and tumor purity with *p* value = 0.032 (Figure 5(e)).

### 3.6. Ferroptosis Could Facilitate the Polarization of M1 Macrophages in Cervical Cancer

From the bioinformatical analyses of the correlation of ferroptosis with tumor-infiltrating immune cells, it could be clearly seen that the proportion of M1 macrophages became obviously higher in the low-risk group. In order to further verify the relevant results, we excogitated the influence of ferroptosis on M1 macrophage polarization using real-time-qPCR. The outcomes illustrated that the expression amount of TNF-*α*, which is the marker towards M1 macrophages, got significantly elevated in M1 macrophages, as a consequence of coculturing M1 with SiHa cells (treated with erastin the day before) (Figure 5(f)). To further verify the conclusion, we conducted ELISA test with the supernatant extracted from coculture dishes, indicating that TNF-*α* secretion level increased gradually with the increasing amount of ferroptosis inductor (Figure 5(g)). From the above analyses and experimental verification, it could be clearly demonstrated that ferroptosis could promote the polarization of M1, therefore creating a better immune microenvironment for cervical cancer patients.

### 3.7. CC Patients Can Be Divided into Subgroups with Different Survival Level Based on Ferroptosis Marker Expression Pattern by NMF

**Figure 6:**
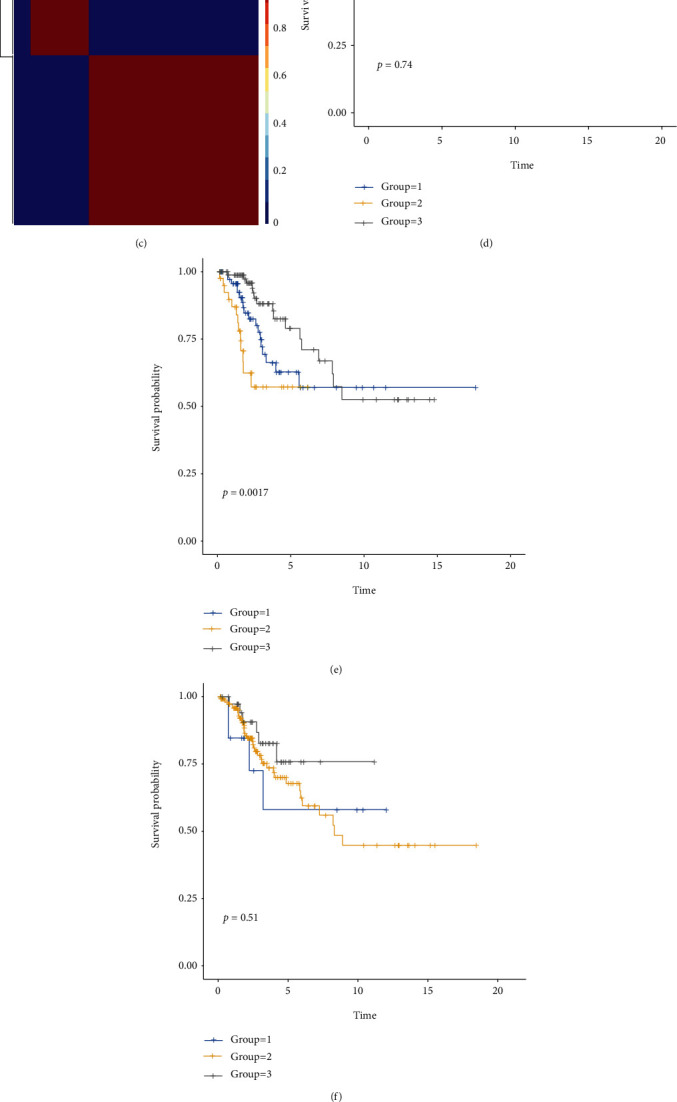
(a)–(c) The consensus maps depicting the outcomes of NMF clustering on the gene expression matrix, in the order of ferroptosis driver, marker, and suppressor, respectively, sharing the same optimal *k* − value = 3. For all the 3 figures, patches from left to right represented groups 1-3. (d)–(f) Kaplan-Meier survival curves of subgroups created by NMF-clustering. From (d) to (f), they are corresponding to driver, marker, suppressor matrix. Considering *p* < 0.05 as significance, Marker gene is the only gene group that matters to OS. (g)–(i) The significance of mRNA expression after erastin induction was PTGS2, VEGFA, and CXCL2 from high to low.

NMF clustering was conducted on the submatrix of expression partitioned according to the function of genes for ferroptosis. Every time we divided the samples into optimal numbers, the survival rate will be compared between all the subgroups. It turned out to be a “marker” function-set that showed significant survival differences (Figures 6(a)–6(f)). In protein-protein interaction analysis on model genes done via Cytoscape software, the PTGS2, CXCL2, and VEGFA ranked relatively high in the topological method DEGREE with their scores > 5 (Supplementary Figure 1, Table 1). Therefore, we narrowed down the range of survival-affecting key genes to which are considered to function as ferroptosis marker among model genes.

### 3.8. Ferroptosis Increased the Expression of PTGS2, VEGFA, and CXCL2 in Cervical Cancer Cells

All the three candidates, PTGS2, VEGFA, and CXCL2, have been proved to correlate with ferroptosis in varying degree. However, the confidence levels of these correlations depend on the methods used to prove them, which differ among these three genes. CXCL2 was noted as “deduced,” while VEGFA “screened,” and PTGS2 “validated.” That is why we need to validate their effectiveness as ferroptosis biomarkers. After treating the SiHa cells with erastin for 24 h, we further detected the expression of PTGS2, VEGFA, and CXCL2. The results showed that ferroptosis could significantly increase the expressing amounts of PTGS2 (*p* value < 0.0001), VEGFA (*p* value < 0.001), and CXCL2 (*p* value < 0.01) in CC cells (Figures 6(g)–6(i)).

### 3.9. Ferroptosis-Marker PTGS2 Affect the Prognosis of Early-Stage CC Patients from Many Aspects

It is aforementioned that among the three markers PTGS2 had the strongest ability to report ferroptosis. Besides, the PTGS2 product tends to be one of the key proteins affecting the model function, which was also verified in GSE44001 by Kaplan-Meier survival analysis (Supplementary Figure 2). Patients got sorted as cohorts with the different expressing amounts, with the median value of expression level of PTGS2 as the cut-off, immune-, and mutation-correlated, and functional enrichment analysis was carried out between those high and low expression clusters.

**Figure 7:**
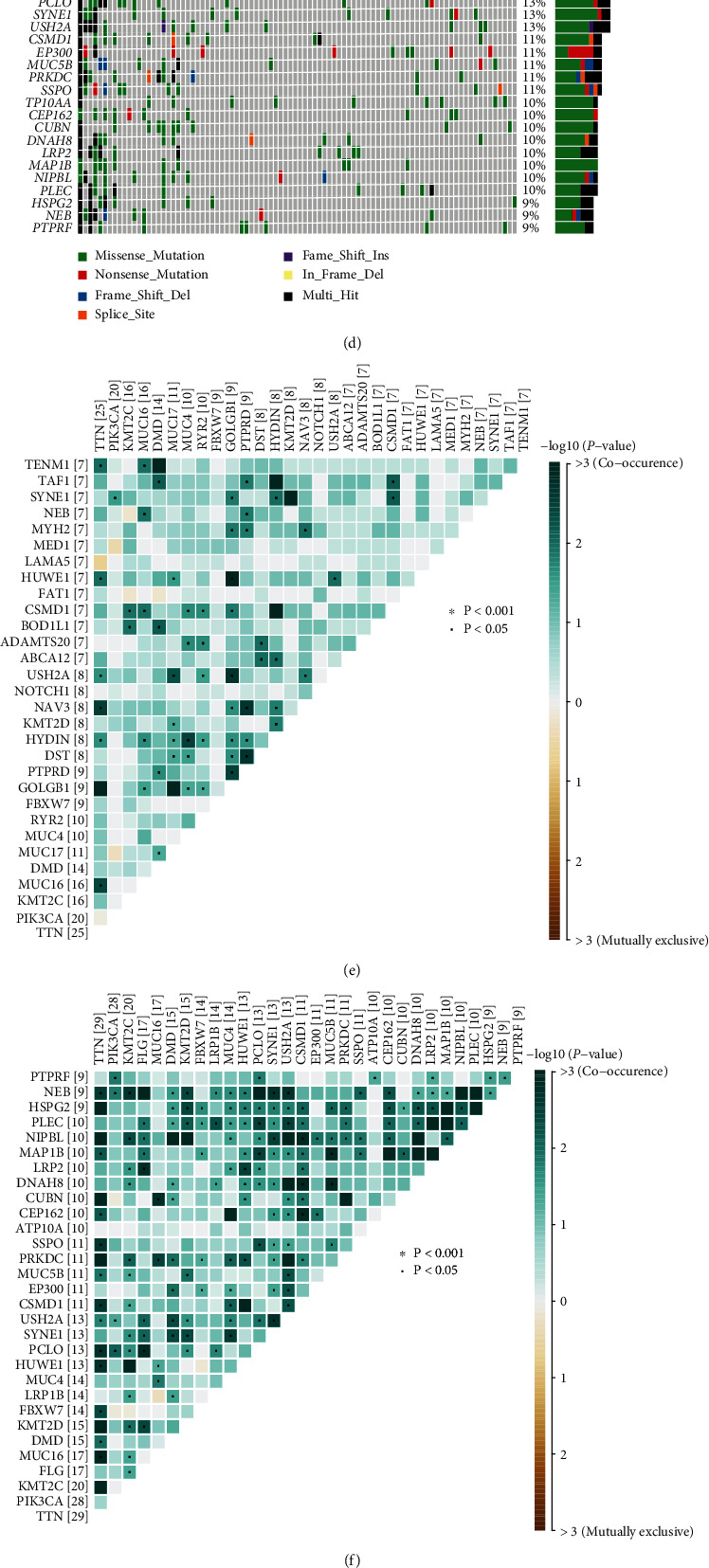
(a) and (b) The TMB distribution differentiates between the clusters with different PTGS2 expression levels, shown in box plot and correlation analysis. (c) and (d) Oncoplots show the mutation spectrums of the top 30 genes with the most quantity of mutation, respectively, in high- (c) and low-PTGS2 (d) groups. The central panels noted the mutated type for each sample. The colored top bar cluster tells the mutation frequency of each sample by providing colored stripes with different lengths. The bottom symptom is noting for mutation types. (e) and (f) The heatmaps demonstrate mutations with the mutual coexistence and exclusion of the top 20 most mutated genes of high- (e) and low-PTGS2 (f) clusters. The color depth of each cell implies the significance of the cooccurring relation. (g) and (h) The forest plot and cooncoplot display the most significantly discrepantly mutated genes. ^∗∗^*p* < 0.01, ^∗^*p* < 0.05, TENM2 and NUP155 rank first in parallel.

**Figure 8:**
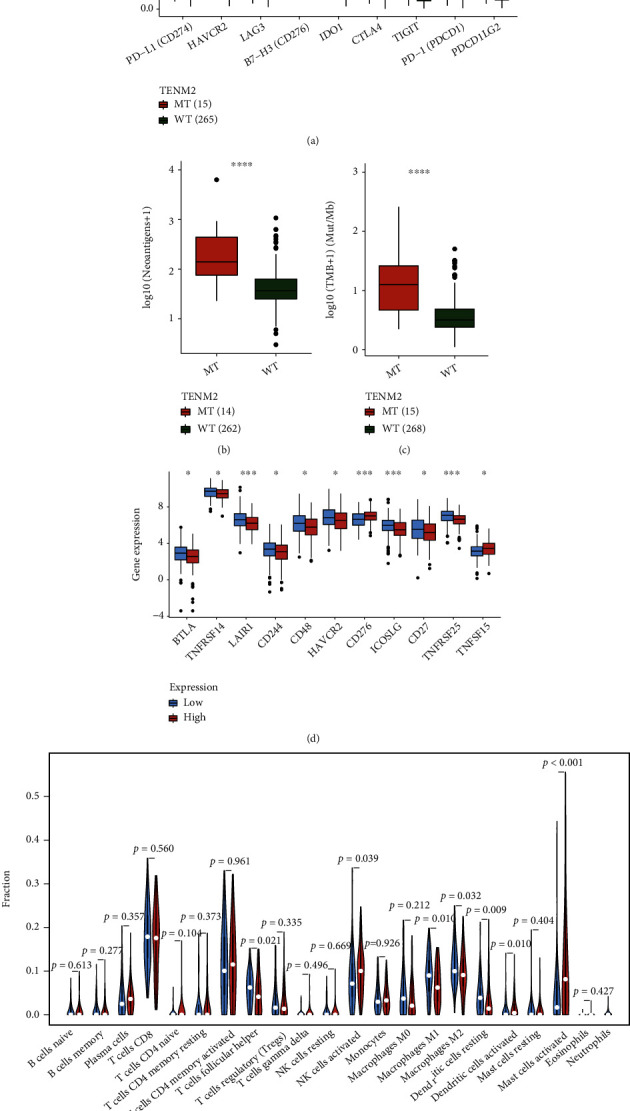
The difference between TENM2 mutant- and wild-type was analyzed via the CAMOIP website. According to checkpoints (a), neoantigens (b), and TMB (c) related analyses, the TENM2 mutated group enjoys a higher expression of PD-1 (PDCD1), neoantigens, and tumor mutation burden, which are all immunotherapy-contributed factors. (d) The ICPs are generally more active in the low-PTGS2 group. (e) Taking *p* value < 0.01 as significant, according to CIBERSORT TIC analysis, the macrophage M1, resting dendritic cells, activated dendritic cells, and activated mast cells show the discrepancy in differently PTGS2-expressed cohorts.

Generally, the low-PTGS2 group carried a greater tumor mutation burden (Figures 7(a) and 7(b)). Somatic mutation data was processed and demonstrated, respectively, in oncoplots and correlation heatmaps. (Figures 7(c)–7(f)). We also filtered out DMGs between two PTGS2-expressed-different cohorts. Ranked in the order of *p* value, the mutations with high significance are overwhelmingly clustered in the low-PTGS2 group (Figure 7(g)). TENM2 and NUP155 rank first in parallel by significance, according to analyses made on drug sensitivity and neoantigens calculated by the CAMOIP website, and the mutant type (MT) of TENM2 is with PD-L1 high-expression, as well as high neoantigens and TMB loads. Features above equipped by TENM2 MT promote the effectiveness of immunotherapy for CC patients, which may contribute to the relatively better survival of the low-PTGS2 group. Genes with the largest quantity of mutations were exhibited in Figure 7(h). Therefore, we carried out a series of analyses to explore if the survival discrepancy can be related to the mutated genes. Inferred from the database from the CAMOIP website, the TENM2 mutant population generally enjoys a higher level of immunotherapy-contributed factors, PD-1 (PDCD1), neoantigens, and tumor mutation burden, for example (Figures 8(a)–8(c)).

ICPs concerning PTGS2 expression were displayed in Figure 8(d). In general, the immune checkpoints seem to express with more activeness in the low-PTGS2 group, except for CD276, whose expression is always found upregulated in malignant tumors and accompanied with a poorer prognosis for cancer patients. Inferred from CIBERSORT scoring, taken *p* value ≤ 0.01 as criteria for significance, macrophages M1, resting and activated dendritic cells, and activated mast cells presented to have a difference on distribution corresponding to PTGS2 expression spectrum (Figure 8(e)). The tendency of macrophage polarization tended to be consistent with that of high and low risk-score clusters, which may account for the result that PTGS2 becomes a determinator to risk degree.

## 4. Discussion

As reported, CC always develops with strong concealment and high mortality. Though researches on the prediction and detection against early cervical cancer had been covered by studies [24–26], few of them were established on the genetic molecular level or concentrated on specific FRGs affecting prognosis in the field of CC. We cut in the theme in the light of the expression information of early CC patients and a prognostic model designed via Cox-LASSO combined methods and based on the gene-expression was constructed.

Ferroptosis, a newfound type of regulated cell death process, has been linked up with cancer treatment from the very beginning of its discovery. In the initial expectation, ferroptosis would be applied as a novel way against the death evasion of cancer cells; however, its effect on tumors presented to be contrary. As an iron-dependent physiological process driven by excessive lipid peroxidation, ferroptosis can be induced by experimental reagents (erastin, etc.), ionization-producing radiation, and cytokines (IFN-*γ*, etc.), concurrently suppressing tumor growth. Yet ferroptosis-resulted damage can also turn on inflammation-associated immunosuppression of TME, favoring tumor growth. The duality also exists in the interaction between the effectiveness of immunotherapy and ferroptosis. cytotoxic-T-cell-driven immunity promotes ferroptotic cell death in cancer cells, which is observed in T cell-driven antitumor immune response launched by immunotherapy with ICIs, mechanistically because of IFN-*γ* launching the JAK-STAT1 pathway and reducing SLC3A2, SLC7A11. PD-L1-targeted antibodies enhance ferroptotic cell death depending on lipid peroxidation, synergistically inhibit tumor growth with ferroptosis activators. Of note, whether there are other cytokines playing the same role as IFN-*γ* and what effect ferroptosis exerts on TME remains unclear, for STAT1 is available for sensitization to a great many ligands. Meanwhile, some kinds of DAMPs (damage-associated molecular patterns) promote tumor growth by accelerating an inflammatory response, which means ferroptosis also contributes to tumor development under specific conditions. HMGB1 released from cancer cells killed by ferroptosis can stimulate inflammatory responses of macrophages when connecting with AGER. According to research about pancreatic cancer, KRAS-G12D emitted inside exosomes by ferroptotic cancer cells can be swallowed by macrophages. Mediated by AGER, this uptake ultimately resulted in an increase of macrophages of M2 phenotype, accelerating tumor growth [27]. So it would seem ferroptosis exerts a long-term effect to do with tumor immunity which depends on immune cell environment, currently discovered inclusive of macrophage polarization and immunoresponse launched by cytotoxic T cell. The comprehensive impact of ferroptosis on tumors can differ under different conditions, and how influential ferroptosis is towards tumor biology remains unknown. Present studies revealed that mutations in specific genes are involved in the response to treatments that activate ferroptosis.

According to bioinformatics analysis carried out to the TME of cohorts with different risk degree divided on model scoring, a significantly different distribution was discovered in macrophage polarization. M1 macrophages enjoyed a larger quantity in samples with better risk degree. Existing researches suggested that M1 is a proinflammatory and antitumor phenotype [28], with the immunophenotype of major histocompatibility complex-(MHC-) II positiveness [29], which means high potential for accelerating inflammatory responses and stimulating the immune system [28]. M1 cells can directly suppress the migration and growth of the tumor as well [30], and these antitumoral effects are also applied in targeted therapies for cancer. Paracrine signals inclusive of TNF-*α* [28], monoacylglycerol lipase [30], TLR [31], NF-*Κ*b [28], and others contribute to the M1 multiplication and antitumor effect [32], so do the cross talks between it and other immune cells; for example, Th1 inducts macrophages towards M1 [30, 33] and the inducible effect of M1 enrichment to the recruitment of CD8+ CTLs [31]. It is reasonable to assume the better prognosis in the low-risk group is related to the difference in macrophage polarization. What is more, M1 macrophages are one of the producers of ferroptosis-inducing cytokines IFN-*γ*, which also participates in the induction of M1 polarity in paracrine and autocrine ways simultaneously [30, 34]. IFN-*γ* signaling mediated by M1 also helped to increase tumor immunogenicity and the presentation of MHC-I on the cancer cell surface, making it more sensitive to cytolysis [35, 36]. Inferred from the above, the regulation of macrophage polarization can be assumed as an unrevealed key clue to the exploration about the effect of ferroptosis on the immune microenvironment of CC patients, and the relatively low mortality of low-risk population may be related to the activation of M1 cells. The prognostic effect of the comprehensive model constructed on FRGs may result from the interaction between ferroptosis and macrophage polarization. It was verified by the result of the following experiment, illustrating that the M1 marker, TNF-*α*, expressed more actively in M1 macrophages, after coculturing SiHa cells treated with erastin and M1 macrophages. It stated with sufficient reasons that ferroptosis affects the polarization of M1 macrophages indeed.

The twenty-five genes constituting the prognostic model were obtained by searching the keyword “ferroptosis” on the GeneCards website and FerrDb database. According to the records in the database, they have different actions in the process of ferroptosis including “driver,” “suppressor,” and “marker,” and the reliability of their association with ferroptosis is also of different levels. Some have been clearly verified by complete experiments, and others come from the conjecture made by researchers with their own knowledge. Among them, there must be core genes of the strongest effects on prognostic prediction. In order to figure out and focus on the key gene, NMF clustering was run three times, respectively, on driver-, suppressor- and marker-gene-expression matrix. It turned out to be the marker group that presented a significant difference in survival rate between its differed gene expression pattern subgroups. PTGS2, CXCL2, and VEGFA are noted as “marker” among the twenty-five, and PTGS2 is the only one with the reliability of “validated” level in accordance with the FerrDb database. Afterward, we treated a group of CC cells with erastin and measured the expression of the candidate genes. All of them showed an increase, and PTGS2 presented to be the most significant (*p* < 0.0001). We thereupon conducted an in-depth analysis around PTGS2.

PTGS2 (prostaglandin-endoperoxide synthase 2) is a gene encoding cyclooxygenase-2 (COX-2). Previous researches show that PTGS2 upregulation is known as a simply downstream marker for ferroptosis and, whether it promotes or suppresses the process of ferroptosis, is still worth searching for the time being. Among 83 oxidative-stress-perturbing genes whose expression levels were surveyed in the experiment, PTGS2 served as the most upregulated candidate that received operation with ferroptosis-induced reagents as erastin or (1S, 3R)-RSL3, and ferroptotic cell death by ferroptosis inductors was not influenced by PTGS-2 suppressor treatment. What is more, PTGS2 upregulation is a consequence of GPX4 loss, also an appropriate biomarker of lipid peroxidation occurring along with ferroptosis [37]. In addition, it is not only an indicator for the result of ferroptosis induction but also with its expression product leading to poor prognosis in CC. In cervical carcinoma, COX-2 and its following product have been found highly expressed [38, 39] and many studies done before showed COX-2 is contributed to carcinogenesis as well as the progression of CC [40]. HPV infection promotes the proliferation of CC cells by mediating the EGFR signal transduction, which was proved to bear upon the acceleration of COX-2 in SCC cell lines [41], and transcription was also affected by oncoproteins E6 and E7 from HPV 16 via EGFR signaling to be concrete [42]. Besides, COX-2 transforms arachidonic acid into prostaglandins in the cytoplasmic membrane [43], with various functions of cell proliferation promotion, E-cadherin running off, loss of cell contact restrain. Such as PGE-2, the major procarcinogenic mediator in the inflammatory environment, acting on surrounding cells by autocrine and paracrine secretion [44]. Thus, there is sufficient theoretical basis for us to determine PTGS2 as hub prognostic-gene and have its expression as an independent risk factor of CC.

Previously, we regarded macrophage polarization as a potential element of how the twenty-five feature model judged the risk of CC patients, with which PTGS2 has also been proved to be in relevance. Previous reports stated that PTGS2 makes the differentiation of macrophages tilt towards M2. In research about diabetic cardiomyopathy (DCM), mesenchymal stem cell (MSC) infusion in rats was prove to induce macrophages towards the M2 phenotype significantly; however, after being pretreated with a COX-2 inhibitor, it failed with this function interestingly, and such consequence could be reversed by prostaglandin E2 (PGE2) adding [45]. Another round goes like, M2-like polarized macrophages turned from human monocytes showed an upregulation of PTGS2, and the coexpression of M2 markers and PTGS2 was found in a specific part of human thyroid tumors. The induction towards M2 by senescent thyrocytes and thyroid tumor cells can be attenuated by COX-2 inhibitors. In thyroid tumors, the relation between COX-2 inhibition and M2 biomarkers downregulation occurred at both early and late tumor stages [46]. It provides a possible explanation for the relative decrease of M1 macrophages in early-stage CC patients when PTGS2 is highly expressed.

It was clearly perceived that TMB and pairs of comutant genes are quantitively much more in the low-PTGS2 group as discussed in Result Section. It might have a bearing on the better efficacy when receiving immunotherapy with high-TMB people. In some other cancer types, high TMB in concert with the expression of PD-L1 was considered as an effective biomarker for ICB therapy, and TMB can be associated with the efficacy of ICB of the combination with ipilimumab and nivolumab alone, where high-TMB tumors are with potential immunogenicity; however, the infiltration and/or activation of T cell is CTLA-4 dependently controlled [47]. Although the conclusion still needs further verification and optimization before being popularly applied according to relative review, the potential possibility for it to affect the survival difference between high- and low-PTGS2 clusters is still worth considering.

## 5. Conclusion

The twenty-five-FRG model can effectively predict the prognosis of CC patients, the inner mechanism of which may be the facilitation from the ferroptosis to M1 macrophage polarization. Among the model genes, PTGS2 turned out to be the hub-gene with its function as a risk factor in cervical cancer. From perspectives of the immune microenvironment and genomic instability, PTGS2 showed its potential on foreshadowing risk, and patients with low-PTGS2 expression will hopefully present a better response to immunotherapy. What is more, the model was designed for patients with early-stage CC, the risk scoring can be referred to by the design of the therapeutic schedule, and may contribute to the early detection of CC, thus promoting prognosis.

## Figures and Tables

**Table 1:** Cervical cancer patients' clinical characteristics distributions.

Characteristic	Levels	Overall
*n*		209
Pathologic stage, *n* (%)	Stage I	149 (71.3%)
	Stage II	60 (28.7%)
Grade, *n* (%)	G1	14 (7.4%)
	G2	95 (50.3%)
	G3	79 (41.8%)
	G4	1 (0.5%)
M stage, *n* (%)	M0	86 (96.6%)
	M1	3 (3.4%)
N stage, *n* (%)	N0	114 (77%)
	N1	34 (23%)
T stage, *n* (%)	T1	118 (67.4%)
	T2	55 (31.4%)
	T3	2 (1.1%)
Human papillomavirus type, *n* (%)	HPV sample	20 (9.7%)
	Normal	187 (90.3%)
Age, median (IQR)		45 (37, 54)

**Table 2:** Primer nucleotide sequence of this study.

Gene	Primer nucleotide sequence
GAPDH	Forward: 5′-CTGGGCTACACTGAGCACC-3′ Reverse: 5′-AAGTGGTCGTTGAGGGCAATG-3′
PTGS2	Forward: 5′-CTGGCGCTCAGCCATACAG-3′ Reverse: 5′CGCACTTATACTGGTCAAATCCC-3′
VEGFA	Forward: 5′-AGGGCAGAATCATCACGAAGT-3′ Reverse: 5′-AGGGTCTCGATTGGATGGCA-3′
CXCL2	Forward: 5′-TGCAGACCGTGCAAGGAATT-3′ Reverse: 5′-TGACCATTCTTGAGAGTGGCTATGA-3′
TNF-*α*	Forward: 5′-GAGGCCAAGCCCTGGTATG-3′ Reverse: 5′-CGGGCCGATTGATCTCAGC-3′

**Table 3:** Information about FRGs from FerrDb of this study.

Characteristic	Driver	Marker	Suppressor
*n*	141	97	94
*Reliability confidence*, *n* (%)			
Deduced	10 (3%)	56 (16.9%)	3 (0.9%)
Predicted	4 (1.2%)	1 (0.3%)	1 (0.3%)
Screened	14 (4.2%)	30 (9%)	1 (0.3%)
Validated	113 (34%)	10 (3%)	89 (26.8%)
*Test subject*, *n* (%)			
Human	61 (18.4%)	77 (23.2%)	49 (14.8%)
Human, mice	29 (8.7%)	1 (0.3%)	33 (9.9%)
Human, mice, rat	0 (0%)	0 (0%)	1 (0.3%)
Human, rat	2 (0.6%)	0 (0%)	1 (0.3%)
Mice	42 (12.7%)	18 (5.4%)	9 (2.7%)
Rat	7 (2.1%)	1 (0.3%)	1 (0.3%)

## Data Availability

The data in support of all the arguments raised in the study have been mentioned in this paper.

## Supplementary Materials

Supplementary Materials Supplementary Figure 1: network shows protein-protein interactions (PPI) among model genes, calculated by DEGREE algorithm. Correlation between genes is represented by straight line, the cycle color represents DEGREE score level, yellow to red from low to high, see Supplementary Table 1 for details of PPI result. In Supplementary Figure 2: seeing from Kaplan-Meier survival analysis between groups with different PTGS2 expression level, divided on the median of PTGS2 expression amounts, low-PTGS2 population enjoy better survivals than high-PTGS2 ones with *p* value =0.032, and samples were collected from GSE44001.
