# Supplementary material for: Identification of a Ferroptosis-Related Prognostic Gene PTGS2 Based on Risk Modeling and Immune Microenvironment of Early-Stage Cervical Cancer
Source: J Oncol. 2022 Apr 8;2022:3997562. doi: 10.1155/2022/3997562 (PMC9012634; doi:10.1155/2022/3997562)
Supplement: Supplementary Materials — Supplementary Figure 1: network shows protein-protein interactions (PPI) among model genes, calculated by DEGREE algorithm. Correlation between genes is represented by straight line, the cycle color represents DEGREE score level, yellow to red from low to high, see Supplementary Table 1 for details of PPI result. In Supplementary Figure 2: seeing from Kaplan-Meier survival analysis between groups with different PTGS2 expression level, divided on the median of PTGS2 expression amounts, low-PTGS2 population enjoy better survivals than high-PTGS2 ones with p value =0.032, and samples were collected from GSE44001. [file 3997562.f1.docx]

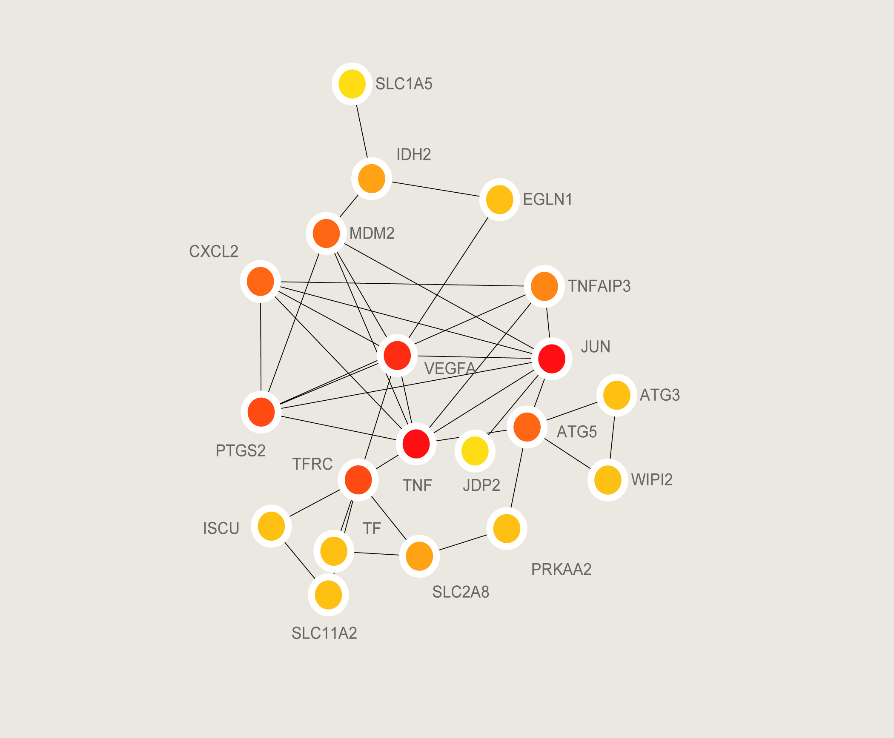


**Supplementary Figure 1.** Network shows protein-protein interactions (PPI) among model genes, calculated by DEGREE algorithm. Correlation between genes is represented by straight line, the cycle color represents DEGREE score level, yellow to red from low to high, seeing **Supplementary Table 1** for details of PPI result.


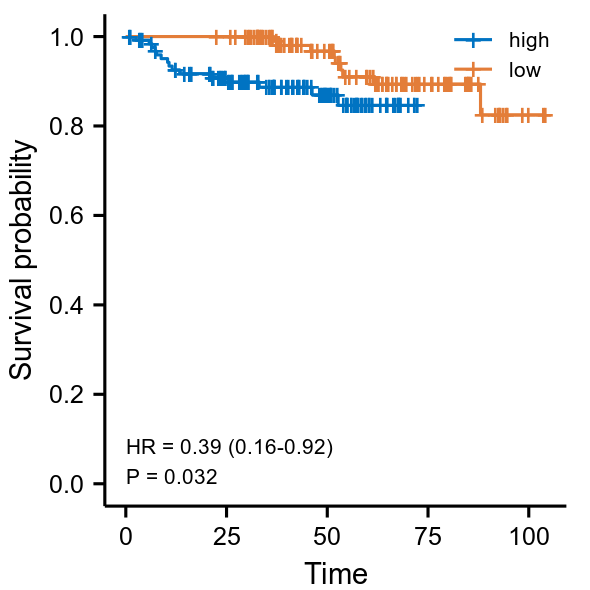


**Supplementary Figure 2.** Seeing from Kaplan-Meier survival analysis between groups with different PTGS2 expression level, divided on the median of PTGS2 expression amounts, low-PTGS2 population enjoy better survivals than high-PTGS2 ones with p-Value = 0.032, samples were collected from GSE44001.

**Supplementary Table1.** Cytoscape results showing model genes with Degree Score > 0

| Rank | Name | Score |
| --- | --- | --- |
| 1 | JUN | 8 |
| 1 | TNF | 8 |
| 3 | VEGFA | 7 |
| 4 | TFRC | 6 |
| 4 | PTGS2 | 6 |
| 6 | ATG5 | 5 |
| 6 | CXCL2 | 5 |
| 6 | MDM2 | 5 |
| 9 | TNFAIP3 | 4 |
| 10 | SLC2A8 | 3 |
| 10 | IDH2 | 3 |
| 12 | SLC11A2 | 2 |
| 12 | ATG3 | 2 |
| 12 | WIPI2 | 2 |
| 12 | PRKAA2 | 2 |
| 12 | TF | 2 |
| 12 | EGLN1 | 2 |
| 12 | ISCU | 2 |
| 19 | JDP2 | 1 |
| 19 | SLC1A5 | 1 |
